# Thermal proteome profiling for interrogating protein interactions

**DOI:** 10.15252/msb.20199232

**Published:** 2020-03-05

**Authors:** André Mateus, Nils Kurzawa, Isabelle Becher, Sindhuja Sridharan, Dominic Helm, Frank Stein, Athanasios Typas, Mikhail M Savitski

**Affiliations:** ^1^ Genome Biology Unit European Molecular Biology Laboratory Heidelberg Germany; ^2^ Faculty of Biosciences EMBL and Heidelberg University Heidelberg Germany; ^3^ Proteomics Core Facility European Molecular Biology Laboratory Heidelberg Germany

**Keywords:** drug discovery, metabolites, protein complexes, proteomics, thermal proteome profiling, Methods & Resources

## Abstract

Thermal proteome profiling (TPP) is based on the principle that, when subjected to heat, proteins denature and become insoluble. Proteins can change their thermal stability upon interactions with small molecules (such as drugs or metabolites), nucleic acids or other proteins, or upon post‐translational modifications. TPP uses multiplexed quantitative mass spectrometry‐based proteomics to monitor the melting profile of thousands of expressed proteins. Importantly, this approach can be performed *in vitro*,* in situ*, or *in vivo*. It has been successfully applied to identify targets and off‐targets of drugs, or to study protein–metabolite and protein–protein interactions. Therefore, TPP provides a unique insight into protein state and interactions in their native context and at a proteome‐wide level, allowing to study basic biological processes and their underlying mechanisms.

## Graphical abstract

This tutorial explains the principles of thermal proteome profiling (TPP) and analyzes the different steps of a TPP experiment. It reviews the recent developments and current applications of this methodology, and provides an outlook of possible new applications.

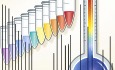

## Introduction

The advent of mass spectrometry‐based proteomics has transformed the study of protein biology, by allowing for a global view of the proteome in its native context (Aebersold & Mann, 2016). This encompasses, for example, the study of protein abundances (Kim *et al*, 2014; Wilhelm *et al*, 2014), turnover (Schwanhausser *et al*, 2011), localization (Geladaki *et al*, 2019), or post‐translational modifications (Potel *et al*, 2018). Recently, biophysical properties of proteins have been explored and studied system‐wide with proteomics approaches.

**Figure 1:**
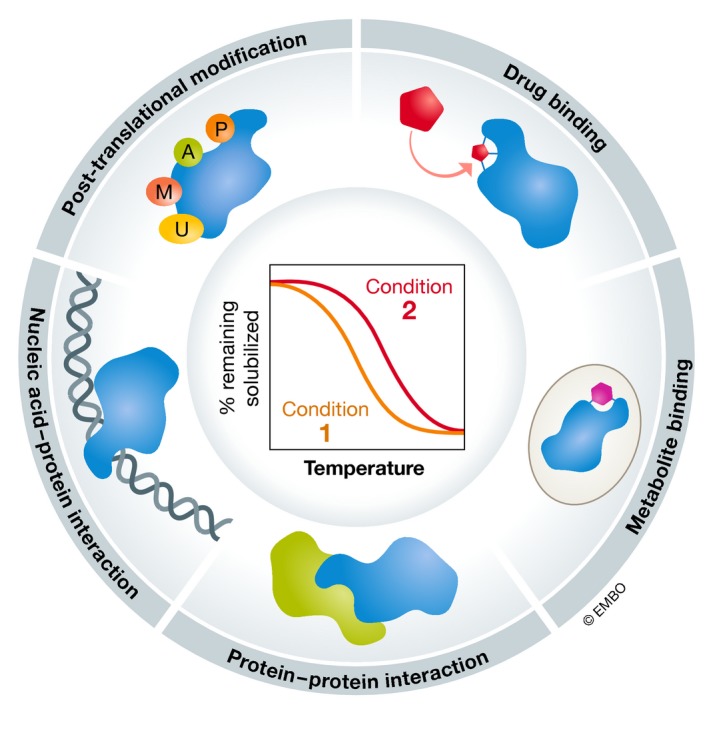
Thermal proteome profiling (TPP) provides proteome‐wide information on protein states and interactions TPP combines the principles of the cellular thermal shift assay (CETSA) with multiplexed quantitative mass spectrometry‐based proteomics. CETSA is based on the principle that proteins denature and become insoluble when subjected to heat. By monitoring the remaining soluble fraction at multiple temperatures, melting profiles for each detected protein can be obtained. The melting profile depends on the context of the protein and can be altered by interactions with small molecules (such as drugs or metabolites), nucleic acids, or other proteins, or post‐translational modifications. CETSA and TPP can be applied *in vitro*,* in situ*, and *in vivo*.

Thermal proteome profiling (TPP; Savitski *et al*, 2014) combines the principles of the cellular thermal shift assay (CETSA; Martinez Molina *et al*, 2013) with multiplexed quantitative mass spectrometry‐based proteomics (Werner *et al*, 2012, 2014). CETSA is based on the long‐standing knowledge that, when heated, proteins denature and generally become insoluble. With CETSA, the heating and aggregation can be performed directly in whole cells, and the soluble protein fraction at each temperature is determined, which allows for generating an *in vivo* melting curve. The melting curve profile is dependent on the context of the protein and can be altered by interactions with small molecules, such as drugs (Martinez Molina *et al*, 2013; Gad *et al*, 2014; Huber *et al*, 2014; Chan‐Penebre *et al*, 2015; Fig 1).

By determining the melting profile of all detected proteins, TPP was initially developed to find targets and off‐targets of drug‐like molecules (Savitski *et al*, 2014, 2018; Huber *et al*, 2015; Reinhard *et al*, 2015; Becher *et al*, 2016; Mateus *et al*, 2016, 2018; Kitagawa *et al*, 2017; Azimi *et al*, 2018; Hu *et al*, 2019; Sridharan *et al*, 2019a)—generally, binding of a drug to a protein leads to a thermal stabilization of the protein (Fig 1). More recently, TPP has been used to identify metabolite‐binding proteins, mapping the proteins which interact with different nucleotides (Huber *et al*, 2015; preprint: Saei *et al*, 2018; Dziekan *et al*, 2019; Sridharan *et al*, 2019b), and unraveling that such interactions can be both promiscuous [e.g., interactions with adenosine triphosphate (ATP)] and very specific [e.g., interactions with thymidine monophosphate (dTMP)]. Binding to nucleic acids also leads to changes in protein thermal stability (Becher *et al*, 2018).

Proteins have also been shown to change thermal stability upon phosphorylation, illuminating the ability of TPP to capture intracellular signaling. For example, inhibition of the BCR‐ABL tyrosine kinase by dasatinib leads to changes in thermal stability of proteins of this signaling pathway, including CRKL (Savitski *et al*, 2014). More recently, direct measurement of phosphorylated proteins has shown that these can display a different melting profile compared to their non‐phosphorylated counterparts (Azimi *et al*, 2018; Huang *et al*, 2019; preprint: Potel *et al*, 2020). Similarly, the redox state of a protein can also alter its melting behavior (Sun *et al*, 2019), and we anticipate that similar stabilization events are yet to be identified for other types of post‐translational modifications.

It was noted early on that kinase inhibitors stabilized not only their kinase targets, but also their tightly interacting regulatory subunits, showing that interacting proteins affect each other's thermal stability (Savitski *et al*, 2014). Indeed, subsequent work has shown that protein complex members tend to have similar melting curves *in vivo*, which has been coined as thermal proximity coaggregation (TPCA) and has been used to monitor protein complex dynamics in their native state in the cell (Becher *et al*, 2018; Mateus *et al*, 2018; Tan *et al*, 2018). Consistently, when a complex is broken apart by genetically removing one of its members (gene knock‐out), the other complex members are thermally destabilized (Mateus *et al*, 2018).

Hence, systematically monitoring changes in protein thermal stability can facilitate the understanding of various cell processes, from that of the downstream effects of drug treatment (Savitski *et al*, 2014, 2018; Huber *et al*, 2015; Reinhard *et al*, 2015; Becher *et al*, 2016; Mateus *et al*, 2016, 2018; Kitagawa *et al*, 2017; Azimi *et al*, 2018; Hu *et al*, 2019) to the detailed study of the eukaryotic cell cycle (Becher *et al*, 2018; Dai *et al*, 2018). This approach can be applied to multiple cellular systems—including lysates, living cells, tissues, or biological fluids—and extends beyond mammalian species. The recent application of TPP to bacteria can expedite the discovery of new antibiotics, by enabling the mapping of the targets of new compounds and understanding of their resistance mechanisms (Mateus *et al*, 2018). New antibiotics are urgently needed in an era in which increasing resistance to existent molecules poses an imminent threat to public health (Brown & Wright, 2016; Tacconelli *et al*, 2018).

We should emphasize that *protein thermal stability* is not correlated with *protein stability*, which is generally described by the protein half‐life (Becher *et al*, 2018; Savitski *et al*, 2018). Nevertheless, there are some links between the two, such as that proteins in complexes have both similar melting curves (TPCA) and similar turnover (Mathieson *et al*, 2018) and that protein clients of HSP90 that require the chaperone throughout their lifetime have lower thermal stability than clients that only require it during synthesis (Savitski *et al*, 2018).

Thermal proteome profiling is part of a larger group of recently developed tools based on proteome stability changes, which include other methods to study heat‐induced protein aggregation (Peng *et al*, 2016; Xu *et al*, 2018), but also methods based on other principles such as the differential proteolytic access upon ligand binding, or changes to protein interactions or conformation, termed limited proteolysis (LiP; Feng *et al*, 2014; Leuenberger *et al*, 2017; Schopper *et al*, 2017; Piazza *et al*, 2018), or the inferring of stability of proteins from rates of oxidation (SPROX; Strickland *et al*, 2013). TPP is so far the only method that allows these types of experiments in living cells. This tutorial is focused on the TPP experimental setup and its recent developments, the multiple data analysis strategies, the current limitations of the methodology, and possible future developments.

## Thermal proteome profiling experimental setup

**Figure 2:**
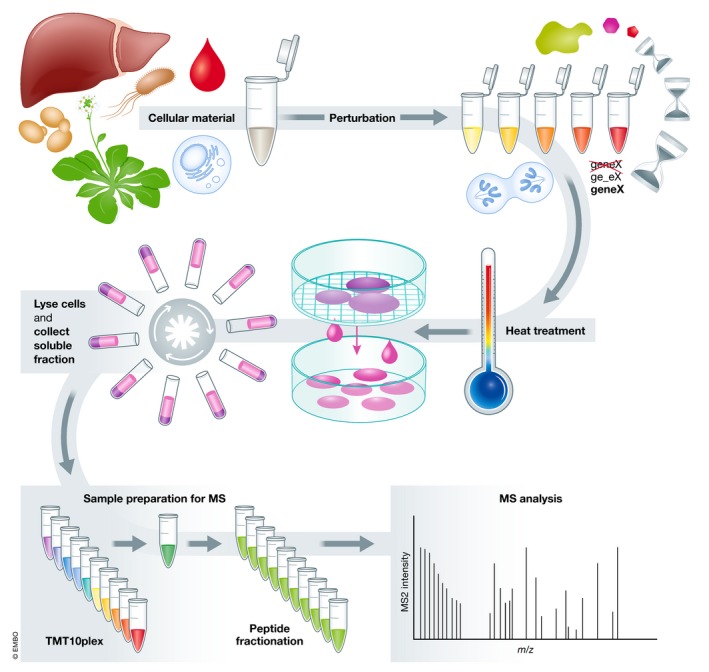
Thermal proteome profiling (TPP) experimental setup (A) TPP starts by the choice of cellular material to study: cell extracts, intact cells, tissues, or biological fluids, from any domain of life (Archaea, Bacteria, or Eukarya, the latter including Protista, Fungi, Plantae, or Animalia). (B) A perturbation can then be induced: commonly a chemical (e.g., drug or metabolite), genetic (e.g., gene knock‐out or overexpression, or point mutation in a gene), environmental, or enzymatic perturbation. Some of these can be applied in a dose‐ or time‐dependent manner. (C) Samples are then subjected to a short heat treatment to induce protein aggregation. (D) The remaining soluble fraction at each temperature is collected after ultracentrifugation or using multi‐well filter plates. (E) Samples are processed using a bottom‐up proteomics workflow, generally using isobaric tandem mass tags (TMT). Labeled peptides are combined and fractionated. (f) Peptides are analyzed by mass spectrometry.

In broad terms, a TPP experiment consists of (i) preparation of the cellular material and induction of perturbation; (ii) heat treatment; (iii) collection of soluble protein fraction; (iv) mass spectrometry‐based proteomic analysis; and (v) data analysis (Fig 2). Step‐by‐step protocols that describe the experiment in detail have been published (Jafari *et al*, 2014; Franken *et al*, 2015). Here, we will highlight the different choices that can be made at each step and detail recent modifications that were not included in the published protocols (Box 1).

### Preparation of the cellular material and induction of perturbation

#### Cellular material

Thermal proteome profiling experiments start by the choice of the biological system to study, i.e., cell extracts, intact cells, tissues, or biological fluids (Fig 2; Box 2).

Cell extracts are prepared by lysis, which dilutes cellular contents (such as proteins, metabolites, and co‐factors) and greatly reduces the normal cell metabolism. Therefore, cell extracts are generally used to identify *direct* targets of perturbations (e.g., the protein(s) to which a drug binds). The extracts can be prepared by mechanical disruption of cells, for example, by douncing (Sridharan *et al*, 2019b) or freeze–thaw cycles (Savitski *et al*, 2014), which can be further aided by enzymatic digestion of certain cell structures [e.g., addition of DNAse to reduce the viscosity of the lysate (Becher *et al*, 2018), or lysozyme or zymolyase to digest the bacterial or yeast cell walls (Mateus *et al*, 2018; Ochoa *et al*, 2019)]. Care should be taken when preparing cell extracts to ensure that proteins remain in their native form—for example, the temperature should not be increased dramatically, and degradation by proteases should be prevented. For the latter, protease inhibitors can be added to the lysis buffer. However, this will prevent observing thermal shifts in these proteins, and therefore, keeping the lysate at low temperature and minimizing the experiment time are generally sufficient to guarantee that proteins are not degraded. The lysates can be clarified by centrifugation to remove insoluble proteins, such as membrane proteins and protein condensates (Savitski *et al*, 2014), although crude lysates have been successfully used (Savitski *et al*, 2018; Sridharan *et al*, 2019b). The latter allow the study of the whole proteome in near native conditions (e.g., preserving most protein complexes and membrane proteins), which has allowed the study of interactions with molecules that cannot enter intact cells, e.g., ATP (Sridharan *et al*, 2019b). The use of detergents to facilitate cell lysis or to solubilize membrane proteins is not recommended at this point, since it has been shown to alter the melting point of proteins (Reinhard *et al*, 2015)—these can be added after the heat treatment, as described below.

Intact cells preserve the physiology of the cell allowing the study of downstream effects of the perturbation (e.g., the (de)activation of a metabolic pathway, or changes in protein levels, or post‐translational modifications). In theory, any cell type can be used, provided that the lysis method does not resolubilize the heat‐induced insoluble protein fraction. To date, the method has been used to profile bacteria (Peng *et al*, 2016; Mateus *et al*, 2018), yeast (Ochoa *et al*, 2019; preprint: Viéitez *et al*, 2019), intracellular parasites (Dziekan *et al*, 2019), plant cells (Volkening *et al*, 2019), or mammalian cells (Savitski *et al*, 2014).

Intact tissues can also be used to preserve the *in vivo* context of cells (Martinez Molina *et al*, 2013; Ishii *et al*, 2017; Perrin *et al*, 2020). These can either be collected and treated with a perturbation, or be collected after the perturbation is performed in the whole organism and systematically analyzed (Perrin *et al*, 2020). This allows the collection of multiple tissues from a single animal, which provides a holistic view of the perturbation in the organism. Biological fluids, such as blood, can also be collected (Perrin *et al*, 2020). In the future, these might offer new therapeutic monitoring strategies or disease biomarkers.

#### Perturbation

Thermal proteome profiling can be applied without any perturbation (other than temperature) to study the melting behavior of proteins *in situ*, unraveling diverse properties of cellular systems, such as that physically interacting proteins have similar melting profiles (Becher *et al*, 2018; Mateus *et al*, 2018; Tan *et al*, 2018).

More commonly, TPP experiments involve chemical [e.g., drug (Azimi *et al*, 2018; Becher *et al*, 2016; Hu *et al*, 2019; Huber *et al*, 2015; Kitagawa *et al*, 2017; Mateus *et al*, 2018, 2016; Reinhard *et al*, 2015; Savitski *et al*, 2014, 2018); or metabolite (preprint: Saei *et al*, 2018; Dziekan *et al*, 2019; Sridharan *et al*, 2019b)], genetic [e.g., gene knock‐out (Mateus *et al*, 2018; Banzhaf *et al*, 2020)], or enzymatic (preprint: Saei *et al*, 2018) perturbations; or different cell states [different phase of the cell cycle (Becher *et al*, 2018; Dai *et al*, 2018), or growth phase (Mateus *et al*, 2018); Fig 2]. Some of the perturbations can be applied in a dose‐dependent manner (Becher *et al*, 2016) or time‐dependent manner (Becher *et al*, 2018; Dai *et al*, 2018) to improve data analysis or facilitate mechanistic understanding of the perturbation (Fig 2). Using this approach, it has been possible to deconvolute drug targets (Savitski *et al*, 2014, 2018; Huber *et al*, 2015; Reinhard *et al*, 2015; Becher *et al*, 2016; Mateus *et al*, 2016, 2018; Kitagawa *et al*, 2017; Azimi *et al*, 2018; Hu *et al*, 2019) and enzyme substrates (preprint: Saei *et al*, 2018), study metabolic shifts (Becher *et al*, 2018; Dai *et al*, 2018; Mateus *et al*, 2018), or identify protein–protein interactions (Tan *et al*, 2018).

### Heat treatment

The next step in a TPP experiment is subjecting the samples to a heat cycle [at a single (Dai *et al*, 2018; Franken *et al*, 2015) or, more commonly, multiple temperatures], which is generally performed in small volumes in a thermocycler, for rapid and homogenous heat transfer (Fig 2). Usually, samples are heated for 3 min, which was initially shown to be sufficient to induce intracellular protein aggregation (Martinez Molina *et al*, 2013). The temperatures should range from a point in which the proteome is not affected, to a point in which the majority of the proteome has become insoluble. Therefore, these need to be adjusted depending on the optimal growth temperature of each organism. The number of temperatures probed is generally limited by practical terms (e.g., analytical capacity or possible range in the thermocycler), although 10 or 12 temperatures with an average of 3–5°C between them have generally been used (a range of 30–50°C). Wider ranges allow the study of larger fractions of the proteome and better interspecies comparisons (Mateus *et al*, 2018), while smaller gaps can detect subtler shifts in melting behavior (Becher *et al*, 2016).

### Collection of soluble protein fraction

After the heat treatment, the remaining soluble fraction at each temperature needs to be extracted (Fig 2). If experiments are performed with intact cells, the cells need to first be lysed. Similar approaches to the ones described above in “Preparation of the cellular material and induction of perturbation” can be used. However, at this point, mild detergents that do not resolubilize the insoluble protein fraction can be used [e.g., NP40 (up to 0.8%), or DDM (up to 1%; Huber *et al*, 2015; Reinhard *et al*, 2015; Hashimoto *et al*, 2018)], which allows monitoring thermal stability shifts in membrane proteins (Reinhard *et al*, 2015). Ultracentrifugation is then used to precipitate the insoluble protein fraction, and generally, the supernatant (soluble fraction) is collected (Savitski *et al*, 2014)—the analysis of the insoluble fraction is also possible, an approach termed target identification by ligand stabilization (TILS), which is claimed by the authors to increase the sensitivity of the method but that has not been further explored (Peng *et al*, 2016). More recently, the soluble protein fraction has been extracted using multi‐well filter plates at low centrifugation speeds, since the insoluble proteins do not traverse the pores of the filter (Mateus *et al*, 2018; Savitski *et al*, 2018). This allows the preparation of large numbers of samples in a benchtop centrifuge and brings TPP to an automatable format that could allow for high‐throughput screens.

### Mass spectrometry‐based proteomic analysis

Protein samples are then processed using a general bottom‐up proteomics workflow, such as in‐gel digestion (Shevchenko *et al*, 2006), in‐solution digestion, filter‐aided sample preparation (FASP; Wisniewski *et al*, 2009), or single‐pot solid‐phase sample preparation (SP3; Hughes *et al*, 2014, 2019; Fig 2). All of these use a protease to digest proteins into peptides (commonly trypsin and/or Lys‐C). The abundance of these peptides in each sample is then quantified by mass spectrometry (Fig 2). Generally, isobaric tandem mass tags (TMT; Werner *et al*, 2012, 2014) have been used to multiplex samples and increase quantification precision (Savitski *et al*, 2014). However, isobaric tags for relative and absolute quantitation (iTRAQ; Ross *et al*, 2004) have also been used (Huber *et al*, 2015), but limit the multiplexing capacity (i.e., fewer temperatures or compound concentrations can be multiplexed), which will generally result in longer analysis time. It is possible that other isobaric labels (Virreira Winter *et al*, 2018; Thompson *et al*, 2019) or even label‐free approaches could also be used.

When samples are multiplexed, they can be combined in multiple ways. In the original approach, now termed TPP temperature range (TPP‐TR), samples from the same perturbation are multiplexed across the multiple temperatures—i.e., each temperature is labeled with a unique isobaric tag and each perturbation results in one sample to be analyzed in the mass spectrometer (Savitski *et al*, 2014; Fig 2). TPP‐TR allows plotting melting profiles, which are essential for the TPCA approach (co‐melting of protein complexes), or can provide additional information about protein interactions. For example, the eukaryotic RNA polymerase II (POLR2A/B) shows a biphasic melting behavior that is only visible in the melting profile, and that reflects the presence of two sub‐populations: one with a higher melting point that is bound to DNA and actively transcribes it, and one that is less thermostable because it is not bound to DNA. The latter is more prevalent during mitosis, when there is a general transcriptional arrest (Becher *et al*, 2018).

When using dose‐ or time‐dependent perturbations, samples from a single temperature can be combined in the same mass spectrometry run—an approach termed TPP compound concentration range (TPP‐CCR; Savitski *et al*, 2014; Franken *et al*, 2015), or if multiple temperatures are analyzed sequentially, two‐dimensional TPP (2D‐TPP; Becher *et al*, 2016; Fig 2). Recently, the 2D‐TPP approach has been extended to discrete perturbations to study the human cell cycle (Becher *et al*, 2018; Dai *et al*, 2018), the effect of gene knock‐outs (Mateus *et al*, 2018; Banzhaf *et al*, 2020), or point mutations (Ochoa *et al*, 2019; preprint: Peck Justice *et al*, 2019; preprint: Viéitez *et al*, 2019). In the 2D‐TPP approach, melting curves for each protein cannot be obtained, since the lowest temperature sample (the reference sample for calculating the remaining soluble fraction at each temperature) is not present in all samples. However, the sensitivity of the method is greatly increased (i.e., it is possible to observe smaller thermal stability effect sizes), since control and perturbation conditions are compared in the same mass spectrometry run. To obtain a melting curve profile while combining treatment and control conditions in the same mass spectrometry run, it is possible to split the probed temperatures across multiple runs. For this, the sample from the lowest temperature is included in all runs (Perrin *et al*, 2020).

It has also been proposed that samples originating from different temperatures of the same perturbation can be mixed prior to multiplexing (effectively, an empirical approach to determine the integral of the melting curve), an approach termed proteome integral stability alteration (PISA) that has the potential to reduce the number of samples analyzed in the mass spectrometer, but is likely to be less sensitive (Gaetani *et al*, 2019).

The mass spectrometry analysis is generally performed on an Orbitrap instrument, since it requires resolving 6 mDa mass differences when using TMT. Quantification of isobaric tags (TMT or iTRAQ) requires the fragmentation of the labels to release the reporter ions that provide the quantification of each condition. If two peptides are co‐isolated for fragmentation, this can lead to a dampening of the expected fold changes, termed ratio compression (Savitski *et al*, 2013). To reduce peptide co‐isolation, pre‐fractionation of the samples with an off‐line chromatographic separation is necessary (Savitski *et al*, 2013, 2018). In addition, MS3 approaches in which peptide fragments are further selected and fragmented can be used (Ting *et al*, 2011; McAlister *et al*, 2014). The MS3 approach increases quantification accuracy, but reduces proteome coverage and precision.

## Thermal proteome profiling data analysis

### Raw mass spectrometry data processing

**Figure 3:**
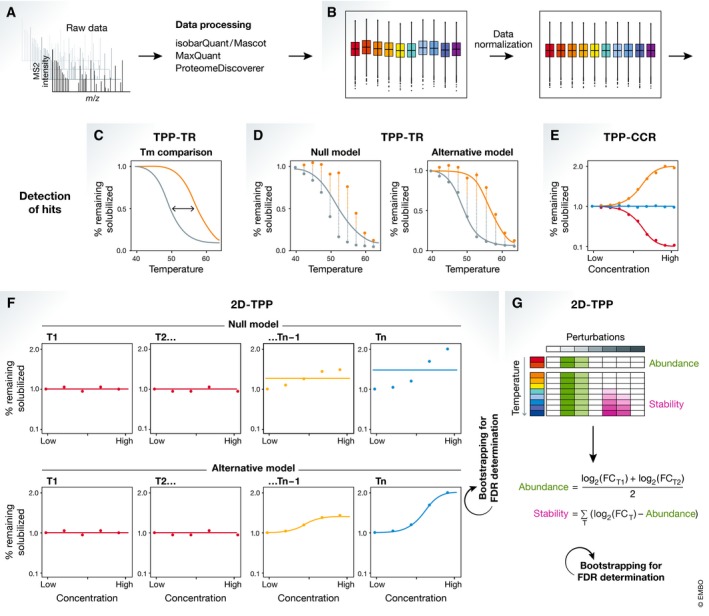
Thermal proteome profiling (TPP) data analysis (A) Raw mass spectrometry data are processed to identify and quantify the measured proteins. (B) Data are then normalized to remove any artifacts introduced during the experimental procedure (e.g., different amounts of protein in each sample). Depending on the type of experiment performed, different analysis strategies exist as follows: (C, D) For TPP‐TR experiments, (C) melting points or (D) whole melting profiles can be compared between conditions. (E) For TPP‐CCR, dose–response curves are fit and targets are selected if a certain degree of stabilization and a good coefficient of determination are obtained. (F) For 2D‐TPP experiments with a dose‐dependent setup, a null model (linear) can be compared to an alternative model (sigmoidal) by comparing the goodness of fit of both models. The false discovery rate (FDR) is inferred by using a bootstrapping approach. (G) For 2D‐TPP with discrete perturbations, a reference condition is selected and fold changes for all other conditions are calculated. To separate abundance from thermal stability effects, this method integrates the relative log‐transformed fold changes measured at the first two temperatures, which are assumed to solely reflect abundance changes. Then, the log‐transformed fold changes are adjusted for the abundance effect and the integral of the log‐transformed fold changes at all temperatures is calculated, which reflect thermal stability changes. In this way, individual perturbations are assigned an abundance and thermal stability score which both are tested for significant deviation from zero by a bootstrapping approach.

The obtained raw mass spectrometry data are processed to identify and quantify the measured proteins. These steps have been usually performed by using isobarQuant (https://github.com/protcode/isob; Franken *et al*, 2015) together with the Mascot search engine (Matrix Science) to identify peptides based on a supplied proteome of the organism used in the experiment (Fig 3A). However, this step can be performed using any proteomics search engine, e.g., MaxQuant (Cox & Mann, 2008) or Proteome Discoverer (Thermo Fisher Scientific).

### Data normalization

Data generally need to be normalized to remove any artifacts introduced during the experimental procedure (e.g., different amounts of protein in each sample due to pipetting errors), which could mask or exaggerate differences between the conditions tested (Fig 3B). Typically, performing a variance stabilizing normalization (VSN; Huber *et al*, 2002; Karp *et al*, 2010) of the reporter ion intensities across replicates of the same treatment conditions but for separate temperatures is recommended. If treatment conditions are expected to vary only in few cases, normalization should be performed across treatment conditions.

Specifically, for TPP‐TR analysis, an additional normalization has been used, since there is a different amount of protein at each temperature. This uses a fit of the medians of relative fold changes per protein profile showing high goodness of fit in each replicate. Then, the parameters obtained from the best fit of median values across replicates are used as reference to obtain normalization coefficients for each replicate. In this way, strong deviations from the expected melting curve can be moderated (Savitski *et al*, 2014; Franken *et al*, 2015).

### Detecting proteins with altered thermal profiles

The most common goal in analysis of TPP datasets is to find proteins with altered thermal profiles between two or more conditions. These conditions can be control and perturbation (one or multiple drug doses, or genetic perturbations), samples originating from different cell types or tissues, or from different physiological states. The analysis approach to find such affected proteins depends on the type of TPP experiment performed (TPP‐TR, TPP‐CCR, 2D‐TPP, or PISA; Box 3).

For TPP‐TR experiments, the first proposed analysis strategy was the comparison of melting points between defined conditions (Savitski *et al*, 2014; Franken *et al*, 2015; Fig 3C). Therefore, melting point estimate differences obtained per replicate (ΔTm) are *z*‐transformed and tested against the null hypothesis of ΔTm = 0. However, reducing the measured data to a single parameter summary statistic comes at the cost of losing sensitivity to detect proteins that show alterations inaccessible by Tm comparison (Childs *et al*, 2019). These include scenarios in which no melting point can be determined—i.e., a given protein does not reach 50% denaturation in the applied temperature range—or the curve differences appear in a different region of the curve, e.g., POLR2A/B which shows stabilization in G1/S vs. M phase only at high temperatures, beyond the melting point. Thus, a new strategy was devised by using of concepts from functional statistics to find altered thermal protein profiles. For this, two models that try to fit the observed data per protein are competed (Fig 3D). The null hypothesis model (μ^0^) fits a smooth function assuming that treatment and control condition follow the same gradually declining curve. On the other hand, the alternative hypothesis model (μ^1^) fits a smooth function separately for each condition. If the μ^1^ model can explain the variance in the observed data better than the μ^0^ model (while being penalized for being able to use more model parameters), a given protein will achieve a high *F*‐statistic: $F=\left({\text{RSS}}^{0}-{\text{RSS}}^{1}\right)/{\text{RSS}}^{1}$, in which RSS represents the residual sum of squares for either the μ^0^ or μ^1^ models. By considering that *F*‐statistics need to be adjusted to meet *F*‐distribution assumptions, these can be converted into *P*‐values, which can be adjusted for multiple testing to control false discovery rate (FDR; Childs *et al*, 2019). Notably, this approach also enables the comparison of multiple conditions. Alternatively, Lim *et al* (2018) have suggested a strategy for TPP‐TR analysis based on integrating curve differences between treatment conditions.

For the analysis of TPP‐CCR experiments, the general strategy involves using the isobaric ratios to fit parametric dose–response curves, and accept targets which show a certain degree of stabilization compared to the no‐drug control (at least 30% or 50%) and exhibit a coefficient of determination (*R* ^2^) surpassing 0.8 (Franken *et al*, 2015; Lim *et al*, 2018; Fig 3E).

The analysis of 2D‐TPP experiments depends on the experimental setup: (i) Treatment conditions used represent a concentration range of a certain treatment, e.g., a small molecule or a tunable perturbation; or (ii) conditions used represent discrete perturbations without an expected dose–response readout.

For the first setup, the initial proposed analysis strategy was similar to the TPP‐CCR approach, with the extra requirement that dose–response effects were observed at multiple temperatures for the same protein (Becher *et al*, 2016). However, this approach suffers from the inability to control the FDR at an *a priori* chosen level. Thus, a new approach was recently developed that employs the same functional analysis concepts from the TPP‐TR approach described above (Kurzawa *et al*, 2019; Fig 3F). This approach competes two models fitting the obtained data under either the hypothesis of no treatment‐induced stabilization, or assuming a dose‐dependent stabilization by the treatment. Comparing the goodness of fit of both models, the method obtains a *F*‐statistic for each protein. Using a parametric bootstrap approach, the FDR is then inferred for each protein, which leads to a more sensitive analysis than the originally proposed method based on fold change and goodness of dose–response fit cutoffs.

In the case in which discrete perturbations are used, a strategy that does not assume a continuous response has been developed (Becher *et al*, 2018; Fig 3G). First, a reference condition is selected [e.g., G1/S phase of the cell cycle (Becher *et al*, 2018) or wild‐type cells (Mateus *et al*, 2018)] to which all other conditions are compared and fold changes are calculated. To separate abundance from thermal stability effects, this method integrates the relative log‐transformed fold changes measured at the first two temperatures, which are assumed to solely reflect abundance changes. Then, the log‐transformed fold changes are adjusted for the abundance effect and the integral of the log‐transformed fold changes at all temperatures is calculated, which reflect thermal stability changes. In this way, individual perturbations are assigned an abundance and thermal stability score which both are tested for significant deviation from zero by a bootstrapping approach, or by using linear models (Ritchie *et al*, 2015).

For the PISA approach, which aims at measuring curve integrals between perturbation and control conditions, a two‐tailed Student's t‐test with subsequent adjustment of *P*‐values for multiple testing can be performed (Gaetani *et al*, 2019).

## Current limitations of thermal proteome profiling

Despite the continuous development of the TPP methodology, several limitations still exist. Some of these are intrinsic to the approach and will be difficult to circumvent. These include the fact that some proteins require extreme temperature conditions that are not practical when the rest of the proteome is also to be monitored [e.g., outer membrane proteins of *Escherichia coli* (Mateus *et al*, 2018)]. Further, some proteins do not perceptibly change in thermal stability upon ligand binding [e.g., BCR‐ABL upon dasatinib treatment (Savitski *et al*, 2014)], and therefore, it is not possible to identify them as perturbation targets. In this case, it is possible to infer them as targets from the downstream effects of their inhibition [e.g., thermal stability shifts of proteins from the BCR‐ABL pathway upon its inhibition by dasatinib (Savitski *et al*, 2014)]. The development of 2D‐TPP has greatly improved the sensitivity of this method; for example, PAH was identified as a target of panobinostat, which was not possible with the TPP‐TR approach (Becher *et al*, 2016).

In contrast, some other limitations will likely be overcome in the near future, such as the lack of detection of low abundant proteins. Further, TPP remains a low‐throughput approach, due to the slow nature of mass spectrometry‐based proteomics. Faster and more sensitive mass spectrometry instruments together with new acquisition modes (Meier *et al*, 2015, 2018) are being developed and allow studying the low abundance region of the proteome. In addition, the possibility of multiplexing up to 16 conditions with isobaric mass tags has been recently realized with TMTpro reagents (Thompson *et al*, 2019). Finally, the cause for the change in melting behavior of proteins cannot be directly inferred from the data, since altered melting behavior can arise from multiple effects to a protein—for example, interactions with small molecules or proteins, or post‐translational modifications. However, TPP can usually narrow the region of the proteome that can be further studied with other methods, and the increasing amount of acquired data might make it possible to train machine learning algorithms to predict the root of thermal shifts.

## Outlook

The TPP methodology has been subject to constant refinements since it was first introduced (Savitski *et al*, 2014), which have increased the sensitivity of the methodology. This includes the addition of a mild detergent to detect thermal shifts in membrane proteins (Huber *et al*, 2015; Reinhard *et al*, 2015), the development of the 2D‐TPP approach (Becher *et al*, 2016), and a way to control for FDR (Kurzawa *et al*, 2019). The adoption of new lysis protocols that have expanded the methodology beyond mammalian cells (Mateus *et al*, 2018; Dziekan *et al*, 2019; Volkening *et al*, 2019) and to intact organs of dosed animals (Perrin *et al*, 2020), the development of new ways to extract the soluble protein fraction for large numbers of samples in parallel (Mateus *et al*, 2018; Savitski *et al*, 2018), and new sample preparation techniques for mass spectrometry (Hughes *et al*, 2014, 2019) have all contributed to increasing the throughput of the procedure.

In the future, TPP might be adapted to an even broader range of applications and might inspire assays for new purposes. Examples are the deconvolution of enzyme‐substrate specificities (preprint: Saei *et al*, 2018) and the recent development of solubility proteome profiling (SPP) for the study of small molecule effects on proteome solubility (Sridharan *et al*, 2019b). The latter was first realized when using the 2D‐TPP approach to identify ATP‐binding proteins in cell extracts. In those experiments, some proteins showed changes at the lowest temperatures, which could only be explained by ATP‐induced changes in protein solubility. Proteins undergo reversible transition from soluble to insoluble state to perform vital cellular functions (Brangwynne *et al*, 2015; Banani *et al*, 2017). Multiple factors, such as protein concentration, metabolites, post‐translational modifications, salt concentration, or temperature, have been shown to influence solubility status of a few recombinant proteins with minimal understanding of the cellular mechanisms that drive these transitions (Mitrea & Kriwacki, 2016). Dysfunction of processes that regulate protein solubility transitions has been suggested as one of the underlying causes for pathological protein aggregation disorders (Aguzzi & Altmeyer, 2016). To this end, the SPP technology enables a proteome‐wide understanding of these solubility transitions by extracting the soluble proteome in the presence and absence of an analyte of interest (e.g., metabolites), as well as, in denaturing conditions (strong detergent for solubilization). SPP makes it possible to study the influence of cellular factors (metabolites, enzymes, etc.), as well as drugs on protein phase transition. Furthermore, it was observed that many proteins that have an insoluble subpopulation under native conditions solubilize upon heating (Sridharan *et al*, 2019b), revealing a classical phase behavior of weakly interacting polymers (Shin & Brangwynne, 2017). Thus, combination of TPP with SPP will be a useful tool to study and establish system‐wide principles of protein solubility transition and to provide an unbiased approach to screen drugs that can prevent aberrant solubility changes.

In summary, TPP is a recently developed tool that provides proteome‐wide information on *in vitro*,* in situ*, and *in vivo* protein states and interactions. This allows studying the mechanisms of a wide range of perturbations and offers new insights into basic biological processes.

## Conflict of interest

The authors declare that they have no conflict of interest.

**Box 1. Nomenclature of different method configurations**

Thermal proteome profiling (TPP) is based on the principles of the cellular thermal shift assay (CETSA) combined with mass spectrometry (MS)‐based proteomics. Therefore, some research groups use the term MS‐CETSA to describe TPP. In this tutorial, the term TPP is used throughout, since that is the term used in the first publication and better captures the proteome‐wide aspect of the technology (Savitski *et al*, 2014).

Some configurations of TPP have gotten specific names to indicate how the samples are multiplexed for mass spectrometry analysis. The original TPP approach (Savitski *et al*, 2014) is now generally termed temperature range TPP (TPP‐TR) to indicate that within the same mass spectrometry experiment, a range of temperatures is multiplexed. During data analysis, these data are represented as melting profiles for each protein. These types of experiments can be used to compare multiple conditions (e.g., drug vs. vehicle, or gene knock‐out vs. wild type). However, it is generally less sensitive than the two‐dimensional approach (2D‐TPP), since the different conditions are analyzed in different mass spectrometry runs. TPP‐TR is the basis of thermal proximity coaggregation (TPCA), i.e., that proteins that interact tend to have similar melting curves.

In the compound concentration range TPP (TPP‐CCR) approach, also introduced in the first TPP publication (Savitski *et al*, 2014), samples from a single temperature, but from multiple compound concentrations are multiplexed. These data are represented as dose–response curves and can be used to estimate compound affinity and rank compounds or targets (Savitski *et al*, 2014).

An extension of this approach is the 2D‐TPP, in which a TPP‐CCR experiment is performed at multiple temperatures (Becher *et al*, 2016). This broadens the list of possible target proteins, since thermal stabilization is generally only observed at temperatures close to the apparent melting temperature (Tm). More recently, this approach has been extended to discrete conditions (e.g., phases of the cell cycle (Becher *et al*, 2018; Dai *et al*, 2018) or gene knock‐outs (Mateus *et al*, 2018; Banzhaf *et al*, 2020)—in which there is not a dose‐dependent response, but each condition is compared to a control).

**Box 2. Choice of cellular material**

The choice of cellular material depends on the aim of the experiment. Cell extracts can be used if the objective is to identify the protein targets of a compound (i.e., the proteins to which a compound binds). Performing the same experiment in intact cells or tissues will provide not only the direct targets, but also any downstream effects of their inhibition (i.e., changes in protein abundance or thermal stability that are the result of the cell responding to the perturbation).

**Box 3. Choice of data analysis method**

The analysis of TPP data depends mostly on the type of experiment performed. For TPP‐TR experiments, either melting points (Savitski *et al*, 2014; Franken *et al*, 2015) or whole melting profiles between different conditions are compared (Childs *et al*, 2019; Fig 3C and D). The latter approach is now preferred, since it allows a broader range of proteins to be analyzed (including those that have atypical melting behavior).

For TPP‐CCR experiments, usually a dose–response curve is fitted and targets are identified as proteins that show a certain degree of stabilization compared to the no‐drug control (e.g., at least 30%), and exhibit a coefficient of determination (*R* ^2^) surpassing 0.8 (Franken *et al*, 2015; Fig 3E).

For 2D‐TPP experiments with a range of concentrations of a compound, a similar analysis to the TPP‐CCR approach can be applied, but requiring protein thermal stabilization at multiple temperatures. Recently, a functional analysis that controls the false discovery rate (FDR) was introduced and is now recommended (Kurzawa *et al*, 2019; Fig 3F).

For 2D‐TPP experiments with discrete perturbations, each condition is compared to a reference condition by calculating fold changes. The changes at the first two temperatures are then used as a proxy for abundance changes, and thermal stability changes are calculated using all temperatures after removal of the abundance effects (Becher *et al*, 2018; Fig 3G).
